# Multispectral drone imagery dataset for plus and non-plus *Neltuma pallida* trees in northern Peru

**DOI:** 10.1016/j.dib.2025.111645

**Published:** 2025-05-09

**Authors:** Wilson Castro, Roberto Seminario, William Nauray, Brenda Acevedo-Juárez, Miguel De-la-Torre, Himer Avila-George

**Affiliations:** a Facultad de Ingeniería de Industrias Alimentarias y Biotecnología, Sullana, 20100, Piura, Perú; b Dirección de Estudios e Investigación, Servicio Nacional Forestal y de Fauna Silvestre, Lima, 150120, Lima, Perú; c Departamento de Ciencias Naturales y Exactas, Universidad de Guadalajara, Ameca, 46600, Jalisco, Mexico; d Departamento de Ciencias Computacionales e Ingenierías, Universidad de Guadalajara, Ameca, 46600, Jalisco, Mexico

**Keywords:** Neltuma pallida, Algarrobo, Deforestation, Multi-spectral image, Plus tree, Seasonally dryed forest

## Abstract

*Neltuma pallida* (“Algarrobo”) is an endangered species native to seasonally dry forests. Preserving this species necessitates creating spatially explicit records of specimens with superior phenological traits, commonly referred to as “plus” trees, which serve as a foundation for reforestation programs. This dataset article describes a collection of multispectral images of Algarrobo trees classified as plus and non-plus, captured between January and September 2023 across the Lambayeque, Piura, and Tumbes departments in northern Peru. Sampling was conducted within forest management zones supervised by the Servicio Nacional Forestal y de Fauna Silvestre. Fieldwork included in-situ evaluation, classification, and georeferencing of specimens, followed by image acquisition using a multispectral drone at a flight altitude of 70 m. Subsequent processing isolated regions of interest corresponding to tree crowns, from which morpho-geometric parameters were extracted, summarized, and compared between tree classes. The dataset contains 500 images per class, geographically distributed across the study area, with plus trees predominantly located in Piura (70 %), Tumbes (20 %), and Lambayeque (10 %). Plus trees exhibit larger mean values for area, perimeter, and major and minor axes. The distributions of major and minor axes and equivalent diameter approximate normal distributions, differing in central tendency and dispersion between classes. However, no significant differences in roundness were observed. This database provides a foundational resource for developing classification models to distinguish between plus and non-plus *Neltuma pallida* trees, supporting conservation and reforestation efforts.

## Specification Table

Table 1 summarizes the main characteristics of the multispectral image dataset of Algarrobo trees.

**Table 1:** Specification table describing the primary attributes of the multispectral image dataset. This table summarizes the subject area, data format, collection procedure, source location, data accessibility, and any related research.

Subject	Agricultural Sciences
Specific subject area	Computer Vision and Pattern Recognition; Agronomy and Crop Science.
Type of data	*.mat
Data collection	The dataset was acquired using a quadcopter drone (Phantom 4 Multispectral, DJI, China). Images were captured across five spectral bands: blue, green, red, red edge, and near-infrared, each with a resolution of 2 MP. The database comprises 500 images per class, captured between 9:00 and 15:00 local time, with the drone flying at a fixed altitude of 70 meters above ground level. Raw images were stored in .TIFF format, organized by location, date, and class. A preprocessing step corrected image displacements and extracted regions of interest (*ROIs*) corresponding to tree crowns. The processed data, including pixel information for the *ROIs*, were saved in *.mat format.
Data source location	**Region:** Tumbes
	**Districts:** Zorrillos, San Jacinto
	**Region:** Piura
	**Districts:** Chulucanas, La Matanza, Veintiséis de Octubre, Sechura, Marcavelica
	**Region:** Lambayeque
	**District:** Lambayeque
Data accessibility	Repository: DRONE_NELTUMA
	doi:10.5281/zenodo.14599251
Related research article	None available.

## 1 Value of the Data

• This database is one of the most extensive publicly accessible collections of multispectral images of individually classified *Neltuma pallida* (Algarrobo) trees in northern Peru. It offers valuable support for forest conservation, species management, and ecological research.
• All regions of interest within the images were manually outlined by forest management specialists. This ensures high-quality segmentation data, making the dataset suitable for developing and benchmarking advanced tree segmentation algorithms.
• Researchers can employ this dataset to build, train, and validate identification models for Algarrobo seed trees using deep learning architectures such as convolutional neural networks. This application has the potential to enhance classification accuracy significantly.
• Morphological parameters derived from this dataset can improve recognition models; however, data augmentation techniques (e.g., rotation, translation) may affect classification performance. Careful experimental design is recommended to mitigate these effects.

## 2 Background

The dataset was developed to provide an open and accessible image resource of *N. pallida* (Algarrobo) trees, captured using unmanned aerial vehicles (UAVs). This resource aims to support research efforts in conservation, forestry management, and machine learning applications.

The images in this dataset were manually classified into plus and non-plus categories by trained personnel based on phenological and morphological traits observed in the field. This manual classification serves as the foundation for the training, validation, and testing of discrimination models. The dataset, stored in .mat format, is particularly well-suited for advanced machine learning algorithms, including Convolutional Neural Networks, facilitating class-based tree crown classification.

Compared to existing resources, this dataset offers significant advantages. It is one of the few publicly available multispectral image collections based on UAVs that are specifically focused on *N. pallida* trees in Peru. In addition, its large sample size and precise classification enhance its utility in developing robust machine learning models, providing a valuable benchmark for future research in forest and computer vision applications.

## 3 Data Description

This paper introduces a dataset comprising multispectral images of *N. pallida* (Algarrobo) trees, acquired using a Phantom 4 RTK multispectral drone. The dataset is organized into two distinct classes: seed trees (“plus”) and non-seed trees (“no plus”), each class containing 500 images stored in .mat format and numbered sequentially.

The dataset is structured for ease of use, with the main folder titled mat_files, which contains two subfolders: files_plus for the plus class and files_noplus for the no-plus class. Both subfolders are compressed into ZIP files to facilitate convenient downloading and distribution. This structure ensures straightforward access for researchers and practitioners working on classification models or related studies.

## 4 Experimental Design, Materials, and Methods

**Fig. 1:**
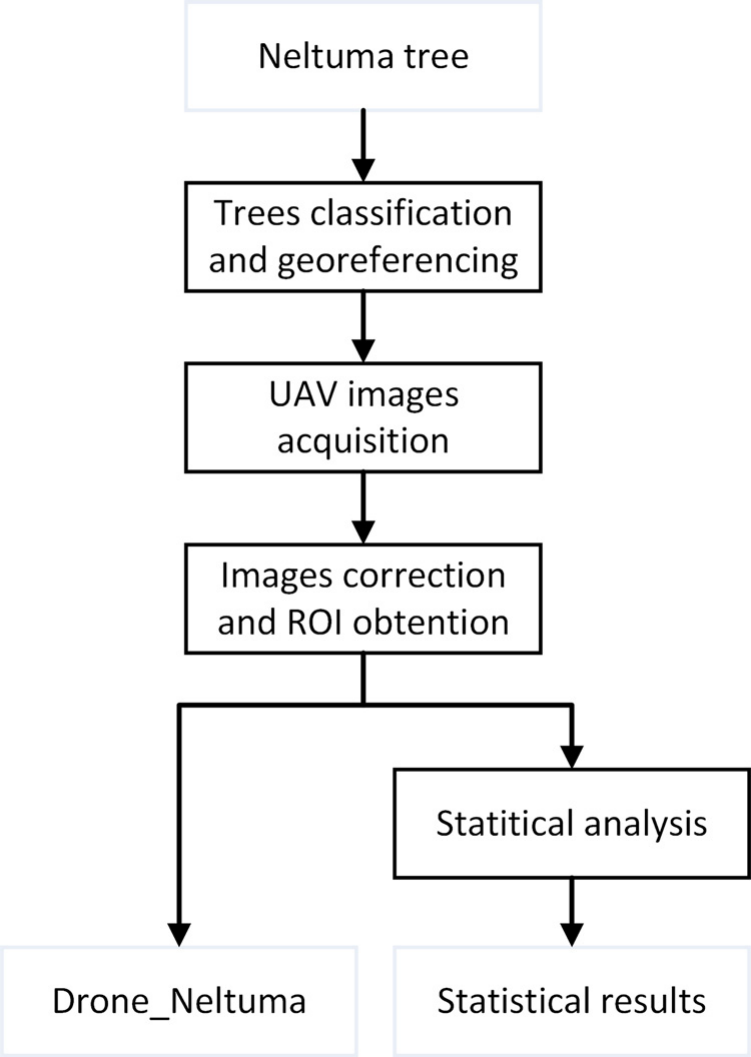
Overview of the methodological procedure for generating the multispectral image database of *Neltuma pallida* trees. This schematic illustrates all major stages, including tree classification and georeferencing, UAV image acquisition, image correction, ROI extraction, and data storage.

The experimental procedure for creating the database is illustrated in Fig. 1. The steps depicted in the diagram are elaborated in detail in the following subsections.

### 4.1 Trees classification and georeferencing

The initial phase consisted of an *in-situ* evaluation of 1000 samples, categorized under the guidance of forestry experts affiliated with the National Forest and Wildlife Service of Peru (SERFOR). The category of *plus* trees included specimens identified as phenotypically superior within their population, possessing one or more characteristics of economic interest as described by Ipinza [1].

**Fig. 2:**
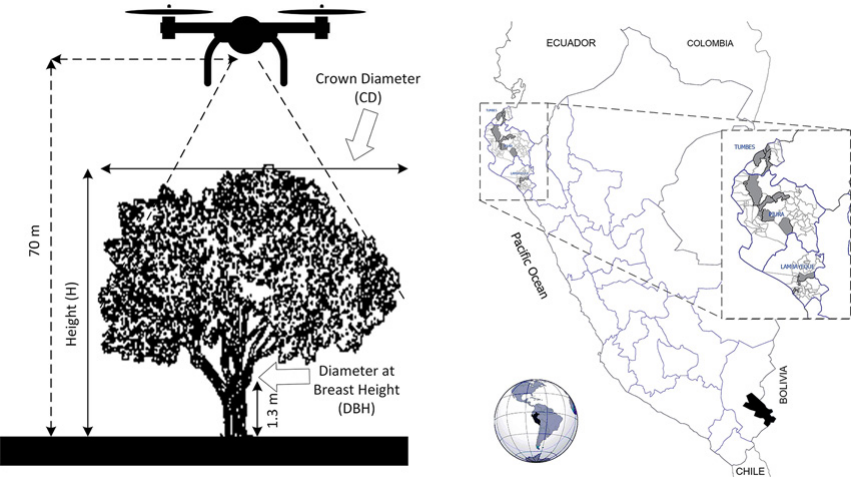
(a) Schematic representation of the UAV-based image acquisition process, including flight altitude and tree parameters. (b) Geographic map of northern Peru showing the distribution of sampled trees in Lambayeque, Piura, and Tumbes.

The minimum criteria for classifying a tree as *plus* were as follows: a trunk diameter at breast height (DBH) of at least 0.45 m, a minimum height from the trunk base to the crown of 13 m, and a crown diameter of 10 m. A diagram illustrating the data collection process and sample images of each tree is presented in Fig. 2(a). Trees that did not meet these criteria were classified as *non-plus*, forming the basis for the training dataset with segmented and labeled images.

The data collection team was deployed to preselected locations (Fig. 2(b)) to implement the *in-situ* collection protocol. The protocol involved measuring samples according to the established criteria and georeferencing each tree. The geographic distribution of the plus trees across the study’s departments is detailed in the accompanying map.

### 4.2 UAV image acquisition

**Fig. 3:**
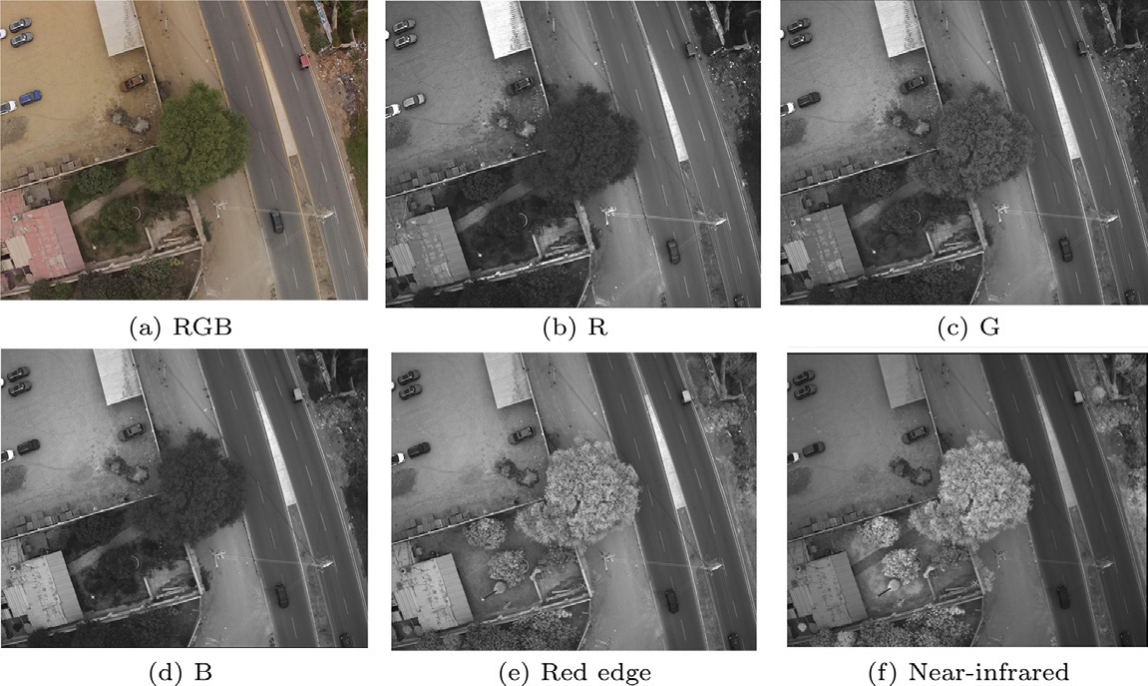
Sample images from the UAV camera: (a) true-color RGB image; (b–f) individual spectral bands for blue, green, red, red edge, and near-infrared. These spectral channels form the basis for subsequent image correction and data analysis.

Flight plans were designed and uploaded to a quadcopter drone (Phantom 4 Multispectral, DJI, China), guided by the geolocations of identified plus and non-plus trees. The drone recorded 2 MP images in five spectral bands (blue, green, red, red edge, near-infrared) and an RGB channel, as depicted in Fig. 3 (please update the figure reference as appropriate).

Following the established protocol, image capture took place between 9:00 and 15:00 local time at a fixed flight altitude of 70 meters above ground level. The resulting dataset is organized by location, with multispectral images stored in .TIFF format and corresponding RGB images stored in .jpg format. Note that any given image may include multiple classes and/or multiple individual trees, owing to the coverage area of each flight.

### 4.3 Image correction and ROI extraction

**Fig. 4:**
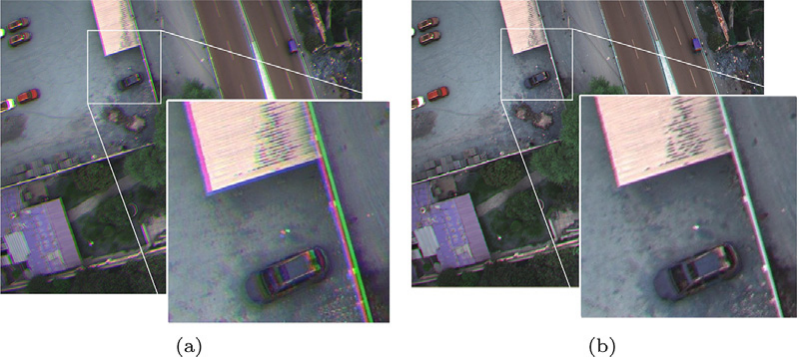
Comparison of (a) an uncorrected RGB image and (b) the same image after translation correction. Note the removal of overlaps among the spectral layers, resulting in improved geometric alignment.

Lens distortion and positional discrepancies were minimized using image translation corrections. This process was applied separately to each of the six bands. An uncorrected RGB image was first generated by concatenating the red, green, and blue bands (Fig. 4(a)). The evaluator then established a reference correction point on the uncorrected image to calculate horizontal and vertical translation distances for each band. These translations were executed using the imtranslate function in MATLAB. A new, fully corrected RGB image was generated, ensuring that no overlapping effects remained among the layers (Fig. 4(b)).

**Fig. 5:**
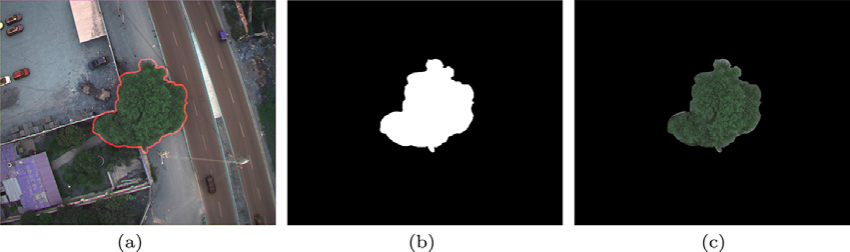
Example of ROI segmentation: (a) freehand mask drawn using MATLABs drawfreehand function, (b) resulting binary mask where pixel values of 1 represent the tree crown and 0 represent the background, and (c) final segmented image highlighting the extracted crown.

After the correction stage, the evaluator manually defined a mask around each tree crown using the MATLAB drawfreehand function (Fig. 5(a)). This mask was stored as a binary image, where pixel values of 1 correspond to the crown and 0 denote the background, see Fig. 5(b). ROI was attached as a sixth layer in the .mat files, coded by department, class, and individual tree number. These comprehensive files facilitate subsequent procedures such as deep learning training or segmentation validation, yielding final segmented images (Fig. 5(c)) suitable for analytical or classification tasks.

### 4.4 Statistical analysis

**Fig. 6:**
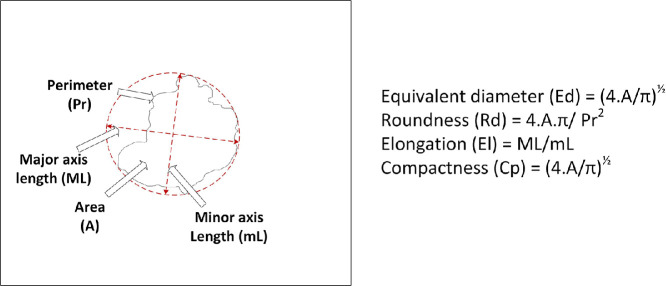
Diagram illustrating the main morphogeometric parameters computed for each segmented crown, including area, perimeter, major axis length, minor axis length, equivalent diameter, and roundness.

The pixel dimensions, determined by the drone’s altitude relative to the tree crowns, were calculated as 0.00376 m/pixel. This resolution was used to compute the fundamental morphometric parameters of the ROIs, as outlined in the works of Mayor et al. [2], Oblitas-Cruz et al. [3] and Oblitas et al. [4]. These parameters are illustrated in Fig. 6.

**Fig. 7:**
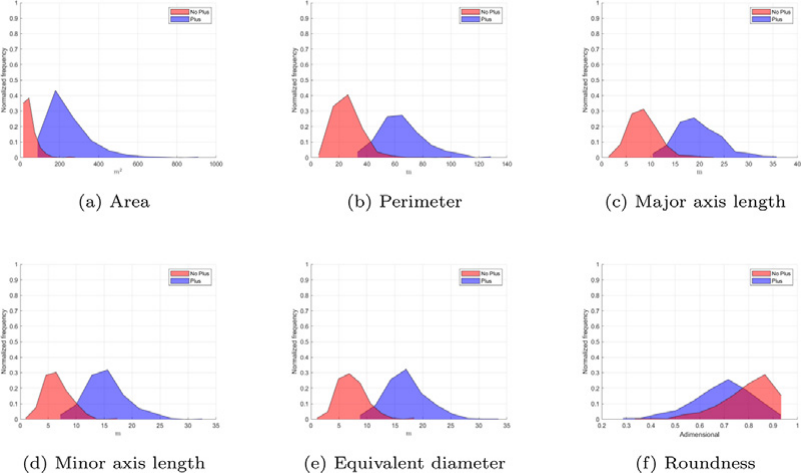
Histograms of crown morphogeometric features for *N. pallida* plus and non-plus trees, including (a) area, (b) perimeter, (c) major axis length, (d) minor axis length, (e) equivalent diameter, and (f) roundness. Each histogram highlights the distribution differences between the two classes.

As shown in Fig. 7(a), the crown area of Plus trees is consistently larger than that of non-plus trees. However, a slight overlap occurs near 100 m^2^, which could serve as an approximate threshold for separating the two classes. Additionally, a few specimens exceed 500 m^2^, representing trees of exceptional size.

A similar trend is evident in Fig. 7(b), where the distributions of both the area and the perimeter exhibit a positive skew. Notably, the perimeter distribution for Plus trees approximates a Gaussian bell curve, with a central tendency around 30 m, whereas the distribution for non-Plus trees centers around 60 m. Despite this, a small overlap between the class distributions is observed, with peaks near 40 m.

Continuing with the analysis of the histograms presented in Fig. 7, similar trends can be observed in Fig. 7(c) to (e), which depict the distributions of the major axis, minor axis, and equivalent diameter, respectively. In these cases, the separation between plus and non-plus tree distributions is more evident, with minimal overlap observed near 15 m for the major axis, 10 m for the minor axis, and 10 m for the equivalent diameter.

The distributions of the major and minor axes, as well as the equivalent diameter, exhibit characteristics resembling a normal distribution, with variations in their central tendency and dispersion. These findings further emphasize that, in all cases (Fig. 7(a) to (e)), the morphogeometric measurements for plus trees are consistently higher than those of non-plus trees.

In contrast, the roundness distributions shown in Fig. 7(f) reveal a less pronounced difference between classes. Although plus trees exhibit slightly higher roundness values compared to non-plus trees, the significant overlap between the two distributions suggests that roundness may not serve as a reliable distinguishing characteristic for classification.

### 4.5 Utility of the database

This database serves as a foundational resource for distinguishing between plus and non-plus *Neltuma pallida* (Algarrobo) trees in seasonally dry forests in northern Peru. Its multispectral nature enables researchers to evaluate and improve segmentation algorithms by benchmarking them against the manually defined ROIs provided by our research team.

In addition, the database supports the development of models based on vegetative indices derived from the spectral bands included (blue, green, red, red edge, and near-infrared). Such indices have been shown to be effective in related studies, such as those conducted by Mwinuka et al. [5] and Yuan et al. [6], demonstrating their potential to advance classification accuracy and forest management applications.

## Limitations

The dataset developed in this study is subject to the following limitations:

• The multispectral sensor is restricted to capturing data in the red, green, blue, red edge, and near-infrared bands. As a result, analysis of crop health and productivity is limited to models that utilize these bands or their combinations.
• The tree classification was based solely on phenological characteristics. Additional production variables, such as pod weight and quality per tree, could provide complementary insights depending on the researcher’s objectives.
• The selection of the ROI, stored as the sixth layer in each file, was performed manually. This process may introduce inconsistencies due to the inclusion of pixels that are not related to the tree. Researchers are advised to use this layer with caution and consider implementing AI-based segmentation models to enhance accuracy.
• External environmental factors, such as wind, cloud cover, and sunlight variations, may have affected the quality of the images by causing positional inaccuracies or motion blur.
• The dataset’s file size results in folders of approximately 9 GB. Consequently, substantial storage capacity and adequate computational resources are required for image processing and analysis.

## Ethic Statement

The authors have read and adhered to the ethical requirements for publication in Data in Brief, confirming that the current work does not involve human subjects, animal experiments, or any data collected from social media platforms.

## Credit Author Statement

**Wilson Castro:** Conceptualization, image acquisition methodology, validation, investigation, resources, funding acquisition and administration, writing - original draft and review. **Roberto Seminario:** Data acquisition and curation, validation, resources. **William Nauray:** Planning of image acquisition routes, design of in situ discrimination methodology, training in the discrimination of plus and non-plus trees, funding acquisition. **Brenda Acevedo-Juárez:** Data validation, writing - original draft and review. **Miguel De-la-Torre:** Software development and validation, formal analysis, data curation, writing - original draft. **Himer Avila-George:** Software development, funding acquisition, writing - original draft, writing - review and editing.

## Data Availability

The multispectral image database is publicly available in ZIP format through the repository at the following doi:10.5281/zenodo.14599251.
